# Locus-specific HLA matching and induction therapy in simultaneous pancreas–kidney transplantation: a national UK cohort study

**DOI:** 10.3389/ti.2026.16427

**Published:** 2026-09-09

**Authors:** Abdullah K. Malik, Jenni Banks, Claire Counter, Lewis Simmonds, Samuel J. Tingle, Sanjay Sinha, Anand Muthasamy, Andrew Sutherland, John Casey, Martin Drage, David van Dellen, Chris J. Callaghan, Doruk Elker, Derek M. Manas, Gavin J. Pettigrew, Neil Russell, Neil S. Sheerin, Colin H. Wilson, Steven A. White

**Affiliations:** 1 Institute of Transplantation, Freeman Hospital, Newcastle upon Tyne, United Kingdom; 2 NIHR Blood and Transplant Research Unit, Newcastle University and Cambridge University, Newcastle upon Tyne, United Kingdom; 3 Newcastle Fibrosis Research Group, Newcastle University, Newcastle upon Tyne, United Kingdom; 4 NHS Blood and Transplant, Bristol, United Kingdom; 5 Oxford University Hospitals NHS Foundation Trust, Oxford, United Kingdom; 6 Imperial College Healthcare NHS Trust, London, United Kingdom; 7 Edinburgh Royal Infirmary, Edinburgh, United Kingdom; 8 Guy’s and St Thomas’ NHS Foundation Trust, London, United Kingdom; 9 Manchester University NHS Foundation Trust, Manchester, United Kingdom; 10 Cardiff and Vale University Health Board, Cardiff, United Kingdom; 11 Cambridge University Hospitals NHS Foundation Trust, Cambridge, United Kingdom

**Keywords:** graft failure, HLA mismatch, induction agent, outcomes, simultaneous pancreas-kidney transplantation

## Abstract

The impact of HLA mismatch and induction therapy in simultaneous pancreas-kidney transplantation on outcomes remains incompletely defined. We conducted a retrospective cohort study of 1705 SPK recipients transplanted in the UK between 2007 and 2019. Using national transplant registry data, we analysed the impact of locus-specific HLA mismatch and induction therapy (Alemtuzumab vs. Basiliximab) on pancreas graft survival primarily. Kidney graft and patient survival were also analysed as secondary outcomes. Multivariable Cox proportional hazards models were adjusted for donor, recipient, and transplant variables. Pancreas graft survival at 1- and 10-year post-transplant was estimated to be 88.6% and 72.7%, respectively. Kidney graft and patient survival at 10 years were 76.7% and 75.3%. In adjusted analyses, donor age, cold ischaemic time, and HLA-DQ mismatch were significantly associated with pancreas graft loss. No significant survival difference was seen between Alemtuzumab and Basiliximab induction therapy. Induction therapy and HLA-mismatch status were not associated with pancreas graft outcome in this large registry study.

## Graphical Abstract

**Figure:**
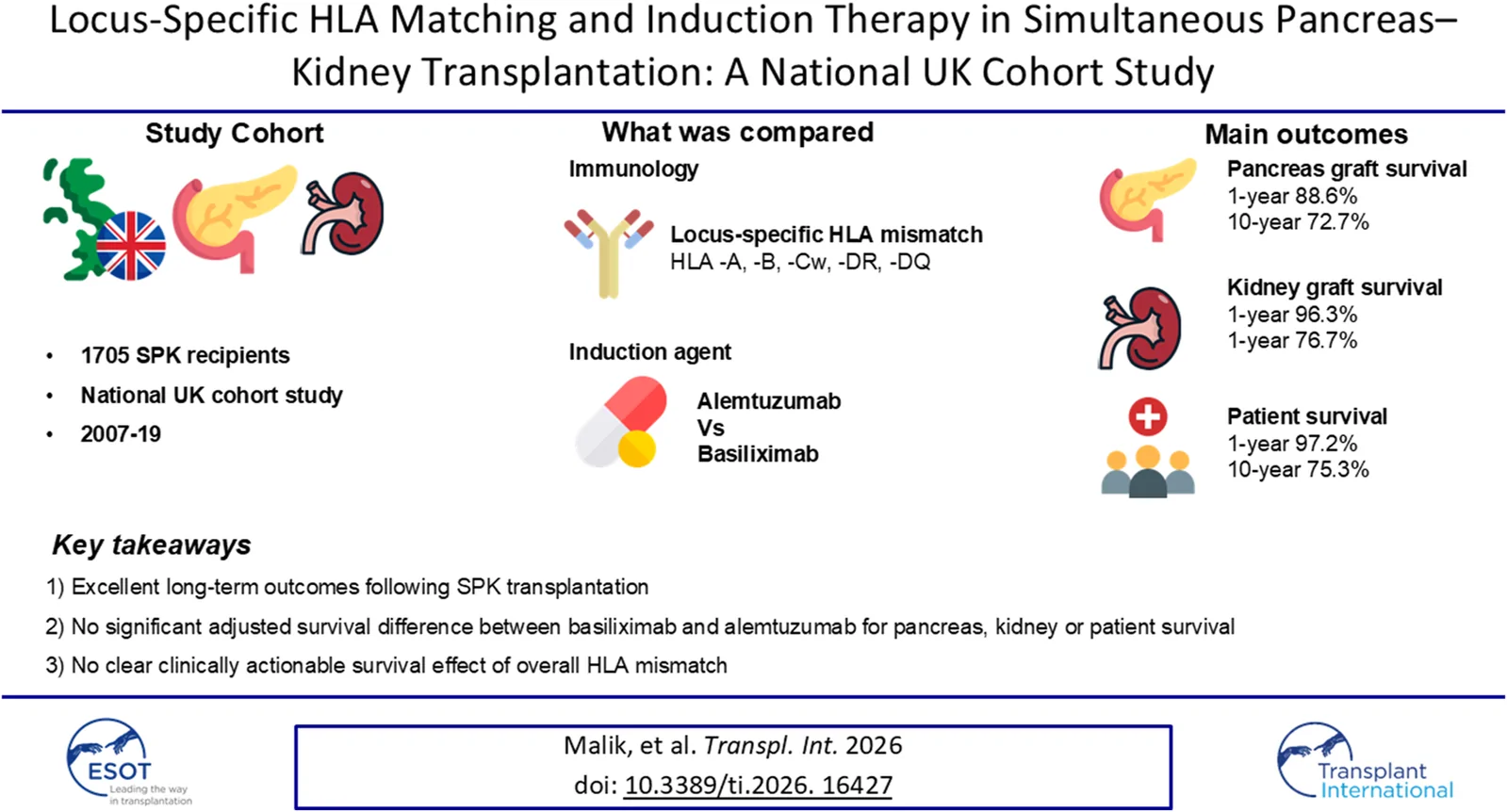

## Introduction

Simultaneous pancreas-kidney (SPK) transplantation is the optimum treatment for selected patients with insulin-dependent diabetes mellitus and end-stage kidney disease, offering superior glycaemic control, renal function and survival compared to dialysis or kidney transplantation alone [1–5]. Recent improvements in immunosuppression, donor and recipient selection, and perioperative care have contributed to enhanced outcomes over recent decades [4, 6–8]. However, long-term pancreas graft survival remains variable, and optimising immunological matching and immunosuppression protocols is warranted to improve graft outcome.

The role of human leucocyte antigen (HLA) mismatching in pancreas transplantation is incompletely understood. HLA matching at the A-, B-, and -DR loci is routine in kidney transplantation [9–11], however the impact of mismatches at the -Cw and -DQ loci is not well studied. Although some studies have suggested that class II mismatches (particularly -DQ) may contribute to *de novo* donor-specific antibody (DSA) development and chronic rejection following deceased donor kidney transplantation [12–14], such associations are not well-established in SPK transplantation. The clinical significance of individual locus-specific mismatches in SPK graft recipients is uncertain [15–17].

In the UK, organ allocation for SPK transplantation incorporates a HLA mismatch component to favour better-matched donor–recipient pairs. Candidates with fewer HLA mismatches receive higher allocation points, which increases their priority for available organs. The system also accounts for recipient sensitisation, quantified as calculated reaction frequency (cRF, analogous to calculated panel-reactive antibody [cPRA] in the United States), such that highly sensitised patients receive additional priority when a compatible donor becomes available. This scoring framework aims to balance immunological compatibility with equitable access for sensitised recipients.

Induction agents administered at the time of graft implantation vary between centres, with Alemtuzumab (a lymphocyte-depleting agent) and Basiliximab (a non-depleting IL-2 receptor antagonist) the commonest agents used. While both agents are widely used in SPK transplantation, direct comparisons of long-term graft and patient survival are lacking, particularly in relation to HLA-mismatch status.

This cohort study investigated the association between HLA-mismatch status and induction agent in terms of long-term outcome following SPK transplantation in the UK. The aim of this study was to inform immunological risk stratification that may potentially guide future allocation and immunosuppression strategies in SPK transplantation.

## Patients and methods

### Setting

This study used data extracted from the UK Transplant Registry (maintained by NHS Blood and Transplant), following approval from the Pancreas Advisory Group. All adult (≥18years) patients who underwent SPK transplantation in the UK from 1st April 2007 to 31st March 2019 were included. Patients who received a normothermic-regional perfusion preserved graft, recipients who underwent re-transplantation, recipients who received an induction agent other than Alemtuzumab or Basiliximab, and recipients with missing outcome data were excluded. One centre exclusively uses anti-thymocyte globulin as their induction agent, therefore all patients from this centre were excluded as numbers were too few for any meaningful analysis.

### Immunosuppression

Induction immunosuppression was with either Alemtuzumab or Basiliximab, according to local centre protocols. Maintenance immunosuppression typically comprised tacrolimus, mycophenolate mofetil, and corticosteroids (tapered according to centre-specific protocols), but detailed maintenance regimens were not uniformly available in the dataset.

### Definitions and outcomes

Demographic and clinical variables included donor and recipient age, sex, ethnicity, blood group, donor type, cytomegalovirus (CMV) status, cause of death, body mass index, creatinine at retrieval, recipient diabetes aetiology, dialysis modality, sensitisation at transplantation, waiting time to transplant, cold ischaemic time, and transplant era (grouped into three time periods by financial year: 2007–2011, 2011–2015, and 2015–2019). HLA mismatches were recorded separately for each of the following loci: A, B, Cw, DR, and DQ. NHS Blood and Transplant defined HLA mismatch groups was also included for comparison (grouped into three categories: Level 1 & 2 due to small numbers of Level 1 transplants, Level 3 and Level 4, Supplementary Table S1).

The primary outcome was pancreas graft survival at 10-year post-transplant. Pancreas graft survival at 1-year post-transplant, and additionally kidney graft survival and patient survival at both horizons were analysed as secondary outcomes. Graft survival was defined as the time from transplant to graft failure, censoring for death with a functioning graft. Patient survival was defined as the time from transplant to patient death. Patients lost to follow-up were censored at the date of last known status.

Acute pancreas graft rejection rates at 3- and 12-month post-transplant were obtained. For follow-up which was ‘Not reported’, it was assumed that no rejection episodes occurred.

### Statistical analysis

Demographic and clinical factors are summarised by induction therapy group to describe the cohort. Categorical variables are presented as counts and percentage, and comparisons between the groups made using Fisher’s exact test or Chi-Squared test. Continuous variables are reported as medians and interquartile range (IQR), and comparisons made using the Student’s t-test.

Kaplan-Meier survival analysis was used to estimate the unadjusted survival for each outcome (pancreas graft, kidney graft and patient mortality). Survival curves were stratified by induction therapy group, and log-rank p-values allowed for comparisons between the groups.

Observations with missing data for the outcome or exposure variables were excluded from the analysis. To retain the sample size when multivariable modelling, missing data for the covariates were addressed using simple imputation. For categorical variables, the most frequently occurring category was imputed while continuous variables were imputed using median values. A comparison of this analysis and a complete-case analysis for the primary outcome was performed.

Separate Cox proportional hazards models were built for each survival outcome (pancreas graft, kidney graft, and patient survival). Each model was stratified by transplant centre to account for centre-specific baseline hazard functions. Stepwise backward selection was used, with variables retained if p < 0.10. Continuous variables were assessed for non-linearity using natural cubic splines and included in that form where appropriate.

A final model was created for each outcome incorporating any covariate found to be significant at any time horizon. The proportional hazards assumption was assessed using cumulative hazard function plots. Model checking involved assessing covariate functional form by Martingale residuals and checking for influential observations using df betas and likelihood displacement. Individual HLA locus models and induction therapy covariates were added to the model to evaluate their significance. The final model for analysis of the primary outcome was assessed for multicollinearity using the Variance Inflation Factor.

Further exploratory analyses of these outcomes at 3 and 5 years were performed to assess consistency with the results at other time horizons; however, these results are not presented in this paper. An interaction term between mismatch at HLA-DQ and induction therapy was included in the multivariable model for the primary outcome to assess potential effect modification. A 1-month landmark survival analysis was performed for the unadjusted pancreas graft survival, excluding any patients who died or were censored within the first month of transplantation.

All analyses were performed using SAS Enterprise Guide version 7.13 (SAS Institute Inc., Cary, NC).

## Results

### Demographics, induction agent and HLA-mismatch status

During the study period, 1897 adult patients underwent first-time SPK transplantation. The final analysis cohort consisted of 1705 SPK graft recipients after excluding recipients transplanted with a graft preserved normothermic regional perfusion (n = 26), recipients who received an induction agent other than Alemtuzumab or Basiliximab (n = 142), and recipients with missing outcome data (n = 24).

The donor and recipient demographic factors for the whole cohort and by induction agent are presented in Table 1 (with further data in Supplementary Table S2). Median recipient age was 43 years (IQR 36–49 years), 720 recipients were female (42.2%), and 70 recipients had Type II diabetes (4.1%). At the time of transplantation, 682 recipients were pre-dialysis (40.0%), and 1,019 recipients (59.8%) were on either haemodialysis (n = 627, 36.8%) or peritoneal dialysis (n = 392, 23.0%). In 1,625 recipients (95.3%), sensitisation was less than 85%. The median waiting time to transplantation was 1.16 years (IQR 0.55–1.17 years), and 1,385 recipients received a graft from a brainstem-death donor (81.2%). Alemtuzumab was used as the induction agent in 1,196 recipients (70.1%) compared to 509 recipients who received Basiliximab (29.9%). The proportion of donation after circulatory death (DCD) graft recipients was greater with Alemtuzumab compared to Basiliximab (20.2% vs. 15.5%, P = 0.025).

**TABLE 1:** Donor and recipient demographics for adult first time simultaneous pancreas and kidney transplants performed in the UK between 1st April 2007 and 31st March 2019.

Factor	Total (n = 1705)	Alemtuzumab (n = 1,196)	Basiliximab (n = 509)	P-value
Donor				
Transplant era (*n, %*)	​	​	​	<0.0001
1 Apr 2007–31 Mar 2011 1 Apr 2011–31 Mar 2015 1 Apr 2015–31 Mar 2019	564 (33.1%) 612 (35.9%) 529 (31.0%)	361 (30.2%) 424 (35.5%) 411 (34.4%)	203 (39.9%) 188 (36.9%) 118 (23.2%)	​
Donor type (*n, %*)	​	​	​	0.025
DBD DCD	1,385 (81.2%) 320 (18.8%)	955 (79.9%) 241 (20.1%)	430 (84.5%) 79 (15.5%)	​
Donor age (years) Median (IQR)	36 (23–47)	38 (24–48)	34 (22–44)	<0.0001
Donor BMI (kg/m^2^) Median (IQR) Not reported (n, %)	23.5 (21.4–25.9) 7 (0.4%)	23.6 (21.5–26.0) 4 (0.3%)	23.2 (21.2–25.5) 3 (0.6%)	0.091
Donor CMV status (*n, %*) Positive Negative Not reported	966 (56.7%) 721 (42.3%) 18 (1.1%)	683 (57.1%) 501 (41.9%) 12 (1.0%)	283 (55.6%) 220 (43.2%) 6 (1.2%)	0.591
Cold ischaemic time (hours) Median (IQR) Not reported (n, %)	11.0 (9.5–13.0) 88 (5.2%)	10.6 (9.2–12.5) 69 (5.8%)	12.0 (10.3–14.0) 19 (3.7%)	<0.0001
Recipient				
Recipient age (years) Median (IQR)	43 (36–49)	43 (36–49)	42 (35–48)	0.005
Recipient sex (*n, %*) Female Male	720 (42.2%) 985 (57.8%)	706 (59.0%) 490 (41.0%)	279 (54.8%) 230 (45.2%)	0.108
Recipient blood group (*n, %*) O A B AB	739 (43.3%) 717 (4201%) 189 (11.1%) 60 (3.5%)	507 (42.4%) 524 (43.8%) 116 (9.7%) 49 (4.1%)	232 (45.6%) 193 (37.9%) 73 (14.3%) 11 (2.2%)	0.002
Recipient cause of diabetes (*n, %*) *Type I* *Type II* *Not reported*	1,468 (86.1%) 70 (4.1%) 167 (9.8%)	1,014 (84.8%) 46 (3.9%) 136 (11.4%)	454 (89.2%) 24 (4.7%) 31 (6.1%)	0.597
Waiting time to transplantation (years) *Median (IQR)* *Not reported* (*n, %*)	1.16 (0.55–1.71) 1 (0.1%)	1.17 (0.54–1.71) 0 (0.0%)	1.16 (0.55–1.71) 1 (0.1%)	0.479
Recipient sensitisation (*n, %*) <85% >85%	1,625 (95.3%) 80 (4.7%)	1,136 (95.0%) 60 (5.0%)	489 (96.1%) 20 (3.9%)	0.382

HLA mismatch distribution varied across loci (Table 2). At the HLA-A locus, 14.8% of recipients were fully matched, 59.8% had one mismatch, and 25.5% had two. For HLA-B, most recipients had either one (47.2%) or two mismatches (48.3%), with complete matches in only 4.5%. HLA-Cw matching was similar: 13.6% had no mismatches, 50.0% had one, and 36.4% had two. A single HLA-DQ mismatch occurred in 58.9%, whereas 34.8% had no mismatches and 6.3% had two mismatches. At the HLA-DR locus, 9.0% were fully matched, while 56.8% and 34.2% had one or two mismatches, respectively. Mismatches were similar across induction agent groups.

**TABLE 2:** HLA mismatches in the whole cohort, and by induction agent.

HLA mismatches	Total (n = 1705)	Alemtuzumab (n = 1,196)	Basiliximab (n = 509)	P-value
HLA-A (*n, %*) 0 1 2	252 (14.8%) 1,019 (59.8%) 434 (25.5%)	187 (15.6%) 707 (59.1%) 302 (25.3%)	65 (12.8%) 312 (61.3%) 132 (25.9%)	0.311
HLA-B (*n, %*) 0 1 2	77 (4.5%) 804 (47.2%) 824 (48.3%)	55 (4.6%) 571 (47.7%) 570 (47.7%)	22 (4.3%) 233 (45.8%) 254 (49.9%)	0.704
HLA-Cw (*n, %*) 0 1 2	231 (13.6%) 853 (50.0%) 621 (36.4%)	163 (13.6%) 595 (49.8%) 438 (36.6%)	68 (13.4%) 258 (50.7%) 183 (36.0%)	0.942
HLA-DQ (*n, %*) 0 1 2	593 (34.8%) 1,004 (58.9%) 108 (6.3%)	408 (34.1%) 705 (59.0%) 83 (6.9%)	185 (36.4%) 299 (58.7%) 25 (4.9%)	0.248
HLA-DR (*n, %*) 0 1 2	153 (9.0%) 969 (56.8%) 583 (34.2%)	95 (7.9%) 694 (58.0%) 407 (34.0%)	58 (11.4%) 275 (54.0%) 176 (34.6%)	0.059
HLA mismatch level (*n, %*) Level 1 and 2 Level 3 Level 4	86 (5.0%) 550 (32.3%) 1,069 (62.7%)	54 (4.5%) 394 (32.9%) 748 (62.5%)	32 (6.3%) 156 (30.7%) 321 (63.1%)	0.243

### Pancreas graft, kidney graft, and patient survival

The unadjusted estimated pancreas, kidney, and patient survival were high over the study period. Pancreas graft survival was 88.6% (95% CI 87.0%–90.0%) at 1-year post-transplant and 72.7% (95% CI 70.1%–75.0%) at 10 years. Kidney graft survival was estimated to be higher with rates of 96.3% (95% CI 95.3%–97.1%) and 76.7% (95% CI 74.2%–79.1%) at 1 and 10 years respectively. Patient survival followed a similar trend, with estimated survival rates of 97.2% (95% CI 96.2%–97.9%) at 1 year, and 75.3% (95% CI 72.6%–77.8%) at 10 years. Most of the graft losses and deaths occurred in the first year following transplantation, with relative stability thereafter.

### Predictors of pancreas graft outcome and acute pancreas graft rejection

**FIGURE 1:**
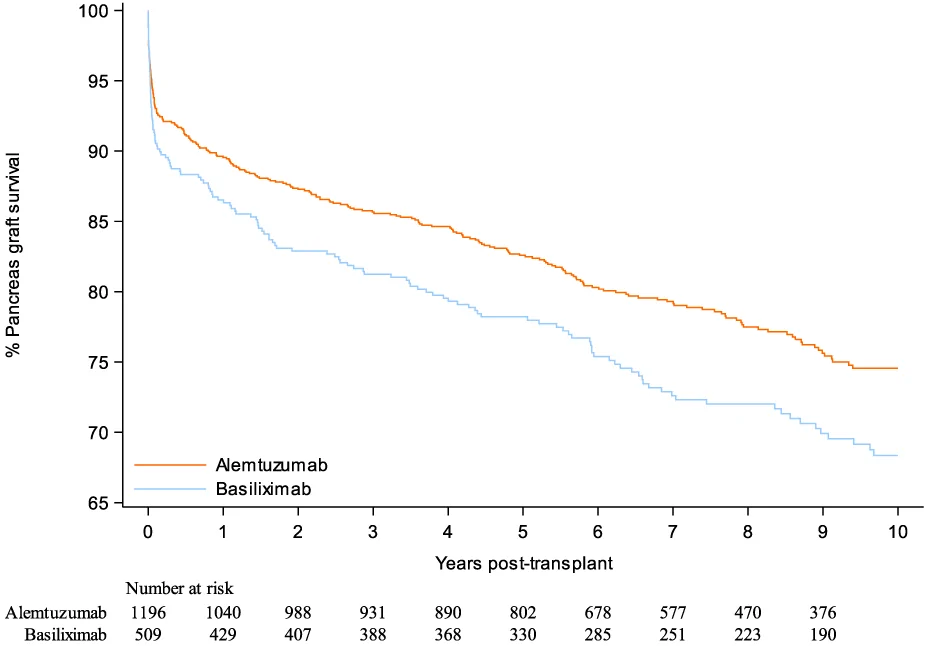
Kaplan-Meier plot of pancreas graft survival at 10-year post-transplant comparing recipients who received Alemtuzumab induction agent with recipients who received Basiliximab.

Unadjusted pancreas graft survival, based on induction agent, is presented in Figure 1, with some evidence to suggest significantly better 10-year survival (p = 0.012) in the Alemtuzumab group (74.6%, 95% CI 71.5%–77.3%) compared to Basiliximab (68.4%, 95% CI 63.6%–72.6%). However, after excluding graft failures that occurred in the first 30 days post-transplant, there was no evidence of a difference in long term pancreas graft survival between the groups, as shown in Supplementary Table S3.

Multivariable modelling of predictors of pancreas graft survival at 1- and 10-year post-transplant are presented in Table 3. Several factors were associated with pancreas graft survival at 10-year. Increasing donor age and longer cold ischaemic time were significantly associated with a higher risk of graft failure (p < 0.0001 and p = 0.015 respectively), and older recipient age was associated with a reduced risk of graft failure (p < 0.0001). Donor CMV positivity was associated with increased risk of graft loss when compared to CMV negative donors (HR 1.25, 95% CI 1.02–1.53, p = 0.030). A sensitivity analysis for handling missing data is presented in Supplementary Table S4, comparing the 10-year pancreas graft survival model estimates for the simple imputation cohort and a complete-case cohort, but the pattern of results was similar between the cohorts.

**TABLE 3:** Multivariable Cox regression model estimates for predictors of pancreas graft survival at 1- and 10-year post-transplant.

Factor	One-year pancreas graft failure		Ten-year pancreas graft failure	
	HR (95% CI)	P*-*value	HR (95% CI)	P*-*value
Era of transplant	​	0.35	​	0.038
Apr 2007-Mar 2011	Reference	​	Reference	​
Apr 2011-Mar 2015	0.81 (0.56–1.15)	​	0.81 (0.64–1.03)	​
Apr 2015-Mar 2019	0.76 (0.51–1.13)	​	0.70 (0.52–0.93)	​
Donor type	​	0.14	​	0.36
DBD	Reference	​	Reference	​
DCD	1.33 (0.91–1.93)	​	1.14 (0.87–1.49)	​
Donor age (years)	1.03 (1.01–1.04)	<0.0001	1.02 (1.01–1.03)	<0.0001
Donor CMV status	​	0.36	​	0.03
Negative	Reference	​	Reference	​
Positive	1.14 (0.86–1.53)	​	1.25 (1.02–1.53)	​
Recipient age (years)	0.98 (0.96–1.00)	0.031	0.97 (0.96–0.98)	<0.0001
Recipient blood group	​	Global 0.37	​	Global 0.059
O	Reference	​	Reference	​
A	1.19 (0.88–1.62)	​	1.16 (0.94–1.43)	​
B	0.84 (0.49–1.42)	​	0.75 (0.52–1.08)	​
AB	0.68 (0.25–1.88)	​	0.70 (0.37–1.33)	​
Sensitisation group	​	0.4	​	0.22
<85%	Reference	​	Reference	​
≥85%	0.71 (0.33–1.56)	​	0.72 (0.42–1.22)	​
Cold ischaemic time (hours)	1.07 (1.02–1.13)	0.005	1.05 (1.01–1.08)	0.015
HLA-A mismatches	​	Global 0.5	​	Global 0.65
0	Reference	​	Reference	​
1	1.30 (0.82–2.06)	​	1.11 (0.81–1.51)	​
2	1.16 (0.69–1.95)	​	1.18 (0.84–1.65)	​
HLA-B mismatches	​	Global 0.26	​	Global 0.008
0	Reference	​	Reference	​
1	0.87 (0.44–1.75)	​	1.25 (0.74–2.11)	​
2	0.68 (0.33–1.41)	​	0.89 (0.52–1.53)	​
HLA-Cw mismatches	​	Global 0.081	​	Global 0.14
0	Reference	​	Reference	​
1	1.28 (0.77–2.12)	​	1.22 (0.88–1.71)	​
2	1.71 (1.00–2.91)	​	1.42 (0.99–2.03)	​
HLA-DQ mismatches	​	Global 0.33	​	Global 0.033
0	Reference	​	Reference	​
1	0.79 (0.57–1.09)	​	0.75 (0.59–0.94)	​
2	0.73 (0.36–1.48)	​	0.94 (0.60–1.47)	​
HLA-DR mismatches	​	Global 0.76	​	Global 0.52
0	Reference	​	Reference	​
1	0.97 (0.59–1.61)	​	1.10 (0.76–1.59)	​
2	0.86 (0.48–1.52)	​	0.97 (0.64–1.46)	​
Induction agent	​	0.93	​	0.91
Alemtuzumab	Reference	​	Reference	​
Basiliximab	0.97 (0.55–1.72)	​	0.98 (0.66–1.45)	​

Abbreviations: DBD, donor after brainstem death; DCD, donor after circulatory death; CMV, cytomegalovirus; HLA, human leucocyte antigen; HR, hazard ratio; CI, confidence interval.

Regarding immunological factors, HLA mismatches at most loci were not significantly associated with pancreas graft outcomes. However, one HLA-DQ mismatch was associated with improved long-term pancreas graft survival at 10 years (HR 0.75, 95% CI 0.59–0.94, p = 0.033), compared to zero mismatches. This finding was not observed for two DQ mismatches, however the sample size in this category is small (6.3%) presenting high uncertainty in the true effect. HLA-B mismatch status was identified as a predictor of graft loss at 10-year (global p *=* 0.008), however the confidence intervals for one and two mismatches crossed 1.0, therefore the true effect remains uncertain. No significant associations were identified for mismatches at HLA-A, -Cw, or -DR. Induction therapy type (Alemtuzumab vs. Basiliximab) was not significantly associated with pancreas graft survival at 10 years when adjusted for other factors.

An interaction term between mismatch at HLA-DQ and induction therapy was included in the multivariable model for 10-year pancreas graft survival to explore effect modification. There was no evidence of effect modification by induction therapy (p = 0.71) (Supplementary Table S5).

After adjusting for confounders, there were no significant associations between HLA mismatches at any locus and pancreas graft survival at 1 year and induction therapy was also not significantly associated with this outcome, as all estimates were consistent with no effect. At 3-month post-transplantation, there was a non-significant difference in the incidence of at least one acute pancreas graft rejection episode when comparing Alemtuzumab with Basiliximab (6.1% vs. 4.0%, p = 0.06). There was no difference when comparing HLA-B mismatch status (p = 0.34) or -DQ status (p = 0.99). Follow-up data for acute rejection rates at 12 months post-transplant was available for 1,544 (91%) patients, and was no difference when comparing Alemtuzumab with Basiliximab (6.8% vs. 7.5%, p = 0.60), HLA-B mismatch status (p = 0.17) or -DQ status (p = 0.30).

### Predictors of kidney-graft outcome

**FIGURE 2:**
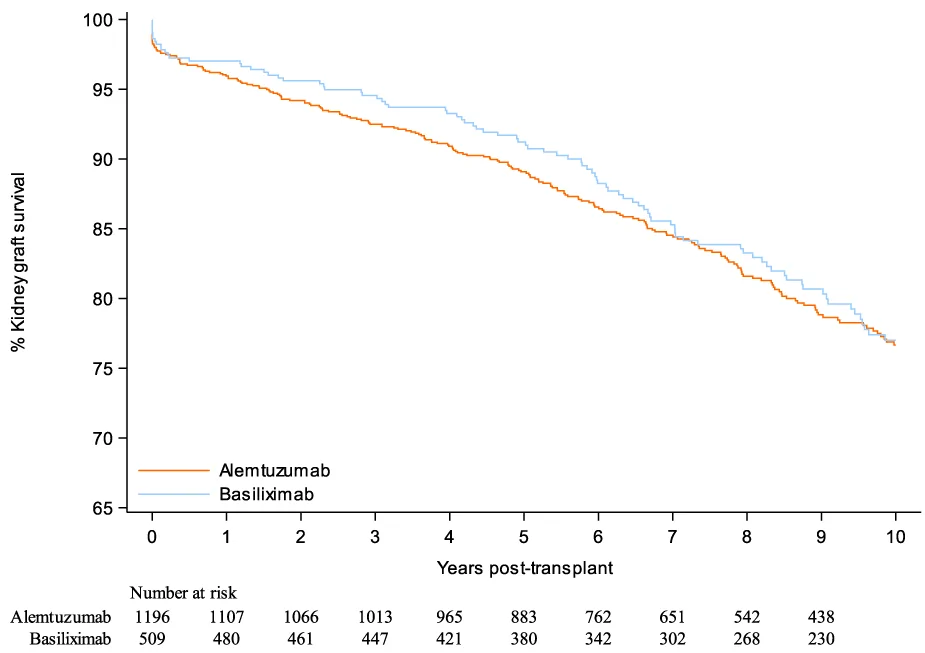
Kaplan-Meier plot of kidney graft survival at 10-year post-transplant comparing recipients who received Alemtuzumab induction agent with recipients who received Basiliximab.

Unadjusted kidney graft survival is presented in Figure 2, with no evidence to suggest a difference in 10-year graft survival (p = 0.601) when comparing Alemtuzumab (76.7%, 95% CI 73.6%–79.4%) with Basiliximab (77.0%, 95% CI 72.3%–81.0%). Predictors of kidney graft survival at 1- and 10-year post-transplant are presented in Table 4. Kidney graft survival improved significantly, with the most recent era (2015–2019) associated with the lowest risk of graft failure at 10 years (HR 0.62, 95% CI 0.44–0.87, p = 0.008). Donor age showed a significant non-linear association with graft loss at 10 years (p < 0.0001). Higher donor creatinine at retrieval was associated with increased graft failure risk at 10 years (HR 1.29, 95% CI 1.07–1.56, p = 0.008). Non-white donor ethnicity and female recipient sex were significantly associated with increased risk of graft failure at 10 years (HR 1.52, 95% CI 1.04–2.22, p = 0.031; and HR 1.46, 95% CI 1.16–1.85, p = 0.002, respectively).

**TABLE 4:** Multivariable Cox regression model estimates for predictors of kidney graft survival at 1- and 10-year post-transplant.

Factor	One-year kidney graft failure		Ten-year kidney graft failure	
	HR (95% CI)	P*-*value	HR (95% CI)	P*-*value
Era of transplant	​	Global 0.0001	​	Global 0.008
Apr 2007-Mar 2011	Reference	​	Reference	​
Apr 2011-Mar 2015	0.25 (0.12–0.51)	​	0.71 (0.54–0.93)	​
Apr 2015-Mar 2019	0.36 (0.18–0.70)	​	0.62 (0.44–0.87)	​
Donor type	​	0.4	​	0.39
DBD	Reference	​	Reference	​
DCD	1.35 (0.67–2.71)	​	1.15 (0.84–1.57)	​
Donor age (years)	Fitted as natural cubic spline	0.042	Fitted as natural cubic spline	<0.0001
Donor creatinine at retrieval (µmol/L)	1.31 (0.85–2.00)	0.22	1.29 (1.07–1.56)	0.008
Donor ethnicity	​	0.92	​	0.031
White	Reference	​	Reference	​
Other ethnic minority	0.95 (0.34–2.69)	​	1.52 (1.04–2.22)	​
Recipient sex	​	0.87	​	0.002
Male	Reference	​	Reference	​
Female	0.96 (0.56–1.63)	​	1.46 (1.16–1.85)	​
Recipient age (years)	Fitted as natural cubic spline	0.13	Fitted as natural cubic spline	<0.0001
Recipient dialysis status	​	Global 0.15	​	Global 0.005
Not on dialysis	Reference	​	Reference	​
Haemodialysis	1.81 (0.99–3.27)	​	1.41 (1.09–1.83)	​
Peritoneal dialysis	1.48 (0.73–2.99)	​	0.89 (0.64–1.23)	​
HLA-A mismatches	​	Global 0.5	​	Global 0.75
0	Reference	​	Reference	​
1	1.60 (0.62–4.15)	​	1.10 (0.77–1.56)	​
2	1.83 (0.67–5.04)	​	1.16 (0.79–1.72)	​
HLA-B mismatches	​	Global 0.99	​	Global 0.66
0	Reference	​	Reference	​
1	0.92 (0.25–3.40)	​	0.78 (0.46–1.33)	​
2	0.95 (0.25–3.60)	​	0.80 (0.46–1.39)	​
HLA-Cw mismatches	​	Global 0.63	​	Global 0.54
0	Reference	​	Reference	​
1	0.89 (0.38–2.08)	​	0.95 (0.66–1.37)	​
2	1.16 (0.48–2.83)	​	1.10 (0.74–1.62)	​
HLA-DQ mismatches	​	Global 0.96	​	Global 0.76
0	Reference	​	Reference	​
1	0.97 (0.53–1.78)	​	0.91 (0.69–1.20)	​
2	0.87 (0.31–2.46)	​	1.00 (0.61–1.63)	​
HLA-DR mismatches	​	Global 0.34	​	Global 0.057
0	Reference	​	Reference	​
1	1.17 (0.42–3.32)	​	0.92 (0.59–1.41)	​
2	1.76 (0.58–5.34)	​	1.26 (0.79–2.02)	​
Induction agent	​	0.8	​	0.74
Alemtuzumab	Reference	​	Reference	​
Basiliximab	0.89 (0.36–2.17)	​	0.92 (0.58–1.48)	​

Abbreviations: DBD, donor after brainstem death; DCD, donor after circulatory death; HLA, human leucocyte antigen; HR, hazard ratio; CI, confidence interval.

Recipient dialysis modality also impacted outcomes, with haemodialysis (vs. no dialysis) significantly associated with worse kidney graft survival at 10 years (HR 1.41, 95% CI 1.09–1.83; p = 0.005). In contrast, peritoneal dialysis was not associated with excess risk when compared to no dialysis at 10 years (HR 0.89, 95% CI 0.64–1.23).

HLA mismatches at all loci were not significantly associated with kidney graft loss at 10 years, when adjusted for other factors. Similarly, induction therapy had no significant impact on kidney graft survival in adjusted models. The same conclusion was observed for 1-year kidney graft survival, as neither the association with HLA mismatch nor induction therapy was significant after adjusting for other factors.

### Predictors of patient mortality

**FIGURE 3:**
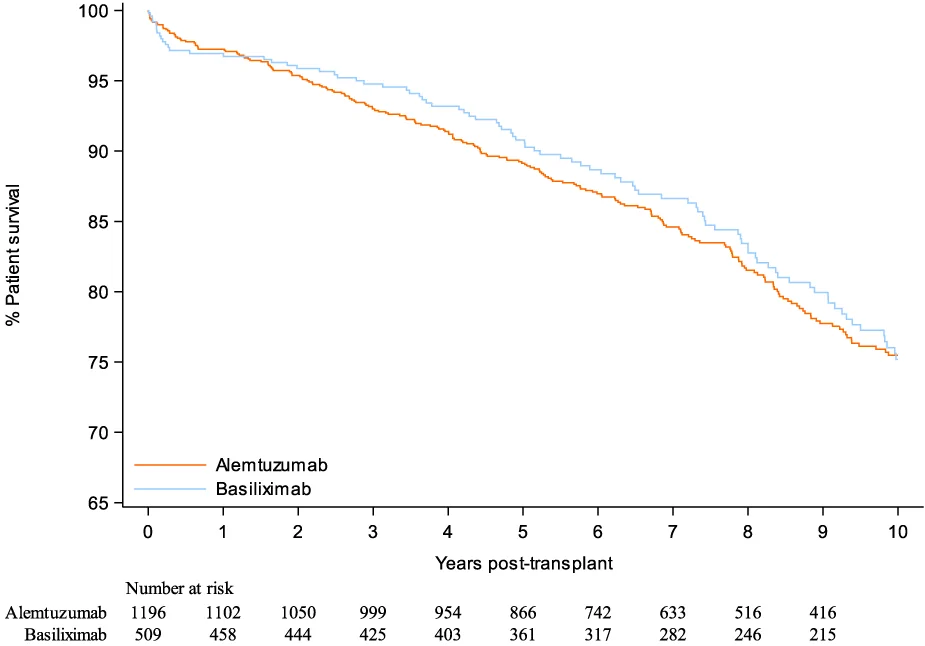
Kaplan-Meier plot of patient survival at 10-year post-transplant comparing recipients who received Alemtuzumab induction agent with recipients who received B.asiliximab.

Unadjusted patient mortality is presented in Figure 3, with no evidence to suggest a difference in 10-year patient survival (p = 0.699) when comparing Alemtuzumab (75.5%, 95% CI 72.3%–78.4%) with Basiliximab (75.2%, 95% CI 70.1%–79.5%). Predictors of patient survival at 1- and 10-year post-transplant are presented in Table 5. Increasing recipient age was a predictor of higher mortality risk with significant association at 10 years (HR 1.04, 95% CI 1.02–1.05, p < 0.0001). Cold ischaemic time was significantly associated with increased 1-year mortality (HR 1.13, 95% CI 1.02–1.24, p = 0.020), but not at 10-year. Longer waiting time to transplant also emerged as a significant non-linear predictor of mortality at 1- and 10-year post-transplant (p ≤ 0.033.) Neither transplant era, donor age, donor CMV status, nor dialysis modality were significant predictors of early mortality.

**TABLE 5:** Multivariable Cox regression model estimates for predictors of patient survival at 1- and 10-year post-transplant.

Factor	One-year patient mortality		Ten-year patient mortality	
	HR (95% CI)	P*-*value	HR (95% CI)	P*-*value
Era of transplant	​	Global 0.23	​	Global 0.5
Apr 2007-Mar 2011	Reference	​	Reference	​
Apr 2011-Mar 2015	0.75 (0.37–1.52)	​	0.96 (0.73–1.28)	​
Apr 2015-Mar 2019	0.45 (0.18–1.12)	​	0.81 (0.56–1.17)	​
Donor age (years)	1.01 (0.99–1.03)	0.34	1.01 (1.00–1.02)	0.053
Donor CMV	​	0.17	​	0.071
Negative	Reference	​	Reference	​
Positive	0.65 (0.35–1.20)	​	1.24 (0.98–1.56)	​
Recipient age (years)	1.02 (0.99–1.06)	0.25	1.04 (1.02–1.05)	<0.0001
Recipient dialysis status	​	Global 0.77	​	Global 0.033
Not on dialysis	Reference	​	Reference	​
Haemodialysis	1.27 (0.64–2.53)	​	1.19 (0.92–1.54)	​
Peritoneal dialysis	1.23 (0.56–2.69)	​	0.76 (0.55–1.07)	​
Cold ischaemic time (hours)	1.13 (1.02–1.24)	0.02	1.01 (0.97–1.05)	0.61
Waiting time (years)	Fitted as natural cubic spline	0.033	Fitted as natural cubic spline	0.019
HLA-A mismatches	​	Global 0.49	​	Global 0.98
0	Reference	​	Reference	​
1	0.67 (0.31–1.45)	​	0.99 (0.71–1.38)	​
2	0.59 (0.23–1.47)	​	1.01 (0.70–1.47)	​
HLA-B mismatches	​	Global 0.73	​	Global 0.95
0	Reference	​	Reference	​
1	0.64 (0.14–3.02)	​	0.96 (0.52–1.77)	​
2	0.79 (0.17–3.78)	​	1.00 (0.54–1.87)	​
HLA-Cw mismatches	​	Global 0.5	​	Global 0.32
0	Reference	​	Reference	​
1	1.94 (0.63–5.95)	​	1.33 (0.90–1.97)	​
2	1.92 (0.59–6.31)	​	1.35 (0.89–2.06)	​
HLA-DQ mismatches	​	Global 0.7	​	Global 0.79
0	Reference	​	Reference	​
1	1.37 (0.65–2.91)	​	1.06 (0.80–1.39)	​
2	1.46 (0.40–5.31)	​	0.91 (0.52–1.58)	​
HLA-DR mismatches	​	Global 0.94	​	Global 0.15
0	Reference	​	Reference	​
1	0.92 (0.28–3.03)	​	0.68 (0.45–1.02)	​
2	1.03 (0.28–3.78)	​	0.75 (0.47–1.17)	​
Induction agent	​	0.92	​	0.7
Alemtuzumab	Reference	​	Reference	​
Basiliximab	1.06 (0.34–3.26)	​	1.09 (0.70–1.69)	​

Abbreviations: CMV, cytomegalovirus; HLA, human leucocyte antigen; HR, hazard ratio; CI, confidence interval.

HLA mismatches at all loci and induction therapy regimen were not significantly associated with patient mortality at either timepoint, when adjusted for other factors.

## Discussion

In this large, contemporary national cohort of SPK transplant recipients, we demonstrate excellent long-term outcomes, with 10-year pancreas graft, kidney graft, and patient survival rates exceeding 70%. Our analysis identifies donor age, cold ischaemic time, and donor CMV status as key predictors of pancreas graft failure and further highlights the adverse impact of haemodialysis on long-term kidney graft survival. Crucially, we identified an unexpected association between a single HLA-DQ mismatch and improved long-term pancreas graft survival, a finding not observed for kidney or patient outcomes. In contrast, induction therapy choice between Alemtuzumab and Basiliximab was not significantly associated with differences in graft or patient survival.

The 3C study, comparing Alemtuzumab with Basiliximab induction therapy in renal transplantation, demonstrated that alemtuzumab significantly reduced the risk of biopsy-proven rejection without increasing serious infections [18]. However, in our study we observed no difference in SPK graft, kidney graft or patient survival on adjusted analyses. This may reflect the distinct immunological challenges of SPK recipients, who may have an alloimmune burden distinct to kidney-only recipients. This also underscores the need for caution in extrapolating induction strategies validated in kidney transplantation to SPK recipients.

The observation that a single HLA-DQ mismatch was associated with improved long-term pancreas graft survival is unexpected and, to our knowledge, has not been previously reported. Although two mismatches were not significantly associated with graft outcome, the overlapping confidence intervals suggests that there is no difference in the effect of two mismatches compared with the effect of a single -DQ mismatch. While class II mismatches, particularly at the DQ locus, have been linked to adverse outcomes in kidney transplantation [12, 19], including increased risk of donor-specific antibody formation and chronic rejection, such associations have not been well established in SPK recipients. This observation could be specific to the model fitted, and not represent true causality warranting cautious interpretation and is, at best, hypothesis-generating. Additionally, the DQ and B mismatches were not associated with acute rejection, suggesting that any observations associated with outcomes may be due to chance rather than a true effect. Further mechanistic investigation using high-resolution HLA typing, epitope analysis, immune profiling, and validation in other populations may provide further insight into the effects of HLA matching on pancreas graft outcomes.

In a retrospective analysis of 1219 pancreas graft recipients (SPK n = 355), HLA mismatch status was not associated with pancreas graft or patient survival following transplantation [17], similar to our results. However, the study reported an increased risk of acute rejection with HLA-B and -DR mismatches with -DQ mismatches having no impact, although this observation was not apparent when SPK graft recipients were examined as a subgroup. These results suggest that the immunological relevance of specific HLA loci may differ by transplant type. These findings suggest that while overall HLA mismatch may not influence long-term outcomes, locus-specific mismatching may contribute to pancreas graft loss through immune mechanisms that are not fully mitigated by standard immunosuppression. This highlights the potential importance of considering HLA matching when evaluating immunological risk, particularly in the context of non-depleting induction strategies, warranting further investigation.

The absence of a clear association between HLA mismatch and kidney graft survival in our cohort may, at least in part, reflect the relatively short cold ischaemia times observed in the UK for SPK grafts. Both HLA mismatch and prolonged cold ischaemia are well-established determinants of kidney graft outcomes, and previous large registry studies have demonstrated that the adverse effect of HLA mismatch is attenuated when cold ischaemia times are short [20, 21]. In our study, the median cold ischaemia time was substantially lower than in comparable international series, likely diminishing the incremental impact of HLA mismatching on kidney graft survival.

Induction therapy did not significantly impact pancreas graft, kidney graft or patient survival on the adjusted analyses. Although unadjusted analyses suggested slightly improved pancreas graft survival with Alemtuzumab, this effect was not observed in multivariable models. The rates of early acute rejection episodes were also comparable between induction agents. Our findings are in line with a retrospective study [22], reporting similar pancreas and kidney graft outcomes between Alemtuzumab and Basiliximab in a large single-centre SPK cohort, though with a higher incidence of CMV infection in Alemtuzumab-treated recipients. More recently, Aziz et al. compared T cell–depleting agents with IL-2 receptor blockade in 417 pancreas transplant recipients and similarly found no difference in graft survival on multivariable analysis [23]. Importantly, both studies highlighted differences in infection profiles rather than survival, with Aziz et al. also noting increased CMV and bacterial infections in the T cell–depleting group. Taken together with our findings, these data suggest that while induction agent choice does not appear to affect long-term graft or patient survival, the decision should be individualised based on recipient risk profile, infection risk, and centre-specific protocols.

Our findings have several potential implications for clinical practice and transplant policy. The absence of a survival benefit from any specific induction agent supports a more individualised approach to induction therapy. Rather than a uniform preference, the choice between T cell–depleting agents and interleukin-2 receptor antagonists may be better guided by recipient comorbidity, CMV serostatus, and institutional experience. Furthermore, we did not observe a significant reduction in graft or patient survival with locus-specific mismatching warranting specific investigation into the organ-specific effects in larger external cohorts.

This study has several limitations inherent to its retrospective design and use of national registry data. While the dataset is robust and population-based, it lacks granularity in certain key areas, including high-resolution HLA typing, DSA status, maintenance immunosuppression regimens, and biopsy-proven rejection. As such, we were unable to explore mechanistic explanations for the observed DQ mismatch effect or evaluate potential interactions between induction, maintenance therapy, and immunological risk. Additionally, selection bias may influence the choice of induction agent, although our multivariable analyses aimed to adjust for relevant confounders. Finally, while the finding of a protective association with a single DQ mismatch is statistically robust and biologically plausible, it is hypothesis-generating and requires validation in other cohorts with complementary mechanistic data.

This national study demonstrates excellent long-term outcomes following simultaneous pancreas–kidney transplantation and provides new insights into the impact of immunological matching and induction therapy. The absence of significant differences in outcomes by induction agent supports a personalised approach to immunosuppression. Although the associations described are observational, and causality cannot be deterimined in this registry study, these findings highlight the complex interplay between immunogenetic matching and induction therapy in SPK transplantation. Our results emphasise the importance of tailoring immunological risk assessment beyond aggregate mismatch scores.

## Data Availability

The data analyzed in this study is subject to the following licenses/restrictions: The data is held by NHS Blood and Transplant, and may be available upon reasonable request. Requests to access these datasets should be directed to statistical.enquiries@nhsbt.nhs.uk.
