# Supplementary material for: Locus-specific HLA matching and induction therapy in simultaneous pancreas–kidney transplantation: a national UK cohort study
Source: Transpl Int. 2026 Sep 9;39:16427. doi: 10.3389/ti.2026.16427 (PMC13598906; doi:10.3389/ti.2026.16427)
Supplement: Supplementary file 1 [file Supplementaryfile1.docx]

Table S1 – NHS Blood and Transplant defined mismatch groups for HLA-A, -B and -DR

| **Level** | **HLA mismatch summary** | **HLA mismatch combinations included** |
| --- | --- | --- |
|  |  |  |
| 1 | 000 | 000 |
| 2 | [0 DR and 0/1 B] | 100, 010, 110, 200, 210 |
| 3 | [0 DR and 2 B] or [1 DR and 0/1 B] | 020, 120, 220, 001, 101, 201, 011, 111, 211 |
| 4 | [1 DR and 2 B] or [2 DR] | 021, 121, 221, 002, 102, 202, 012, 112, 212, 022, 122, 222 |

Table S2 – Full demographics of the cohort

| **Factor** | **Alemtuzumab**  **(n=1196)** | | | **Basiliximab**  **(n=509)** | | | | **Total**  **(n=1705)** | | | | **P-value** | | |
| --- | --- | --- | --- | --- | --- | --- | --- | --- | --- | --- | --- | --- | --- | --- |
| Transplant era |  | | |  | | |  | | | | | | | <0.0001 |
| 1 April 2007 – 31 March 2011 | 361 | (30.2%) | | 203 | (39.9%) | | | 564 | | | (33.1%) |  | | |
| 1 April 2011 – 31 March 2015 | 424 | (35.5%) | | 188 | (36.9%) | | | 612 | | | (35.9%) |  | | |
| 1 April 2007 – 31 March 2011 | 411 | (34.4%) | | 118 | (23.2%) | | | 529 | | | (31.0%) |  | | |
| Donor type |  | |  |  | |  |  | |  | | | | 0.025 | |
| DBD | 955 | (79.9%) | | 430 | (84.5%) | | | 1385 | | | (81.2%) |  | | |
| DCD | 241 | (20.2%) | | 79 | (15.5%) | | | 320 | | | (18.8%) |  | | |
| Donor sex |  | |  |  | |  |  | |  | | | | 0.711 | |
| Male | 594 | (49.7%) | | 258 | (50.7%) | | | 852 | | | (50.0%) |  | | |
| Female | 602 | (50.3%) | | 251 | (49.3%) | | | 853 | | | (50.0%) |  | | |
| Donor blood group |  | |  |  | |  |  | |  | | | | 0.005 | |
| O | 513 | (42.9%) | | 237 | (46.6%) | | | 750 | | | (44.0%) |  | | |
| A | 534 | (44.7%) | | 195 | (38.3%) | | | 729 | | | (42.8%) |  | | |
| B | 116 | (9.7%) | | 70 | (13.8%) | | | 186 | | | (10.9%) |  | | |
| AB | 33 | (2.8%) | | 7 | (1.4%) | | | 40 | | | (2.4%) |  | | |
| Donor CMV status |  | |  |  | |  |  | |  | | | | 0.591 | |
| Negative | 683 | (57.1%) | | 283 | (55.6%) | | | 966 | | | (56.7%) |  | | |
| Positive | 501 | (41.9%) | | 220 | (43.2%) | | | 721 | | | (42.3%) |  | | |
| Not reported | 12 | (1.0%) | | 6 | (1.2%) | | | 18 | | | (1.1%) |  | | |
| Donor ethnic group |  | |  |  | |  |  | |  | | | | 0.315 | |
| White | 1093 | (91.4%) | | 460 | (90.4%) | | | 1553 | | | (91.1%) |  | | |
| Other ethnic minority | 84 | (7.0%) | | 43 | (8.5%) | | | 127 | | | (7.5%) |  | | |
| Not reported | 19 | (1.6%) | | 6 | (1.2%) | | | 25 | | | (1.5%) |  | | |
| Donor cause of death |  | |  |  | |  |  | | <0.0001 | | | | | |
| Vascular | 653 | (54.6%) | | 254 | (49.9%) | | | 907 | | | (53.2%) |  | | |
| Trauma | 119 | (10.0%) | | 96 | (18.9%) | | | 215 | | | (12.6%) |  | | |
| Hypoxic | 308 | (25.8%) | | 113 | (22.2%) | | | 421 | | | (24.7%) |  | | |
| Other / not reported | 116 | (9.7%) | | 46 | (9.0%) | | | 162 | | | (9.5%) |  | | |
| Donor age (years) – median (IQR) | | |  |  | |  |  | | <0.0001 | | | | | |
|  | 38 | (24 – 48) | | 34 | (22 – 44) | | | 36 | | | (23 – 47) |  | | |
| Donor BMI – median (IQR) |  | |  |  | |  |  | |  | | | | 0.091 | |
|  | 23.6 | (21.5 – 26.0) | | 23.2 | (21.2 – 25.5) | | | 23.5 | | | (21.4 – 25.9) |  | | |
| Not reported | 4 | (0.3%) | | 3 | (0.6%) | | | 7 | | | (0.4%) |  | | |
| Donor creatinine at retrieval (mg/dL) – median (IQR) | | | | | |  |  | | |  | | | | 0.097 |
|  | 0.77 | (0.61 – 1.02) | | 0.80 | (0.61 – 1.05) | | | 0.77 | | | (0.61 – 1.03) |  | | |
| Not reported | 1 | (0.1%) | | 2 | (0.4%) | | | 3 | | | (0.2%) |  | | |
| Recipient sex |  | |  |  | |  |  | |  | | | | 0.108 | |
| Male | 706 | (59.0%) | | 279 | (54.8%) | | | 985 | | | (57.8%) |  | | |
| Female | 490 | (41.0%) | | 230 | (45.2%) | | | 720 | | | (42.2%) |  | | |
| Recipient blood group |  | |  |  | |  |  | |  | | | | 0.002 | |
| O | 507 | (42.4%) | | 232 | (45.6%) | | | 739 | | | (43.3%) |  | | |
| A | 524 | (43.8%) | | 193 | (37.9%) | | | 717 | | | (42.1%) |  | | |
| B | 116 | (9.7%) | | 73 | (14.3%) | | | 189 | | | (11.1%) |  | | |
| AB | 49 | (4.1%) | | 11 | (2.2%) | | | 60 | | | (3.5%) |  | | |
| Recipient ethnic group |  | |  |  | |  |  | |  | | | | 0.281 | |
| White | 1055 | (88.2%) | | 442 | (86.8%) | | | 1497 | | | (87.8%) |  | | |
| Other ethnic minority | 130 | (10.9%) | | 65 | (12.8%) | | | 195 | | | (11.4%) |  | | |
| Not reported | 11 | (0.9%) | | 2 | (0.4%) | | | 13 | | | (0.8%) |  | | |
| Recipient sensitisation (%) |  | |  |  | |  |  | |  | | | | 0.382 | |
| < 85 | 1136 | (95.0%) | | 489 | (96.1%) | | | 1625 | | | (95.3%) |  | | |
| ≥ 85 | 60 | (5.0%) | | 20 | (3.9%) | | | 80 | | | (4.7%) |  | | |
| Recipient cause of diabetes |  | |  |  | |  |  | |  | | | | 0.597 | |
| Type I | 1014 | (84.8%) | | 454 | (89.2%) | | | 1468 | | | (86.1%) |  | | |
| Type II | 46 | (3.9%) | | 24 | (4.7%) | | | 70 | | | (4.1%) |  | | |
| Not reported | 136 | (11.4%) | | 31 | (6.1%) | | | 167 | | | (9.8%) |  | | |
| Recipient dialysis status |  | |  |  | |  |  | |  | | | | 0.036 | |
| Haemodialysis | 425 | (35.5%) | | 202 | (39.7%) | | | 627 | | | (36.8%) |  | | |
| Peritoneal dialysis | 295 | (24.7%) | | 97 | (19.1%) | | | 392 | | | (23.0%) |  | | |
| Not on dialysis | 476 | (39.8%) | | 206 | (40.5%) | | | 682 | | | (40.0%) |  | | |
| Not reported | 0 | (-) | | 4 | (0.8%) | | | 4 | | | (0.2%) |  | | |
| Recipient age (years) – median (IQR) | | | |  | |  |  | | |  | | | | 0.005 |
|  | 43 | (36 – 49) | | 42 | (35 – 48) | | | 43 | | | (36 – 49) |  | | |
| CIT (hours) – median (IQR) |  | |  |  | |  |  | |  | | | | <0.0001 | |
|  | 10.6 | (9.2 – 12.5) | | 12.0 | (10.3 – 14.0) | | | 11.0 | | | (9.5 – 13.0) |  | | |
| Not reported | 69 | (5.8%) | | 19 | (3.7%) | | | 88 | | | (5.2%) |  | | |
| Waiting time to transplant (years) – median (IQR) | | | |  | |  |  | | |  | | | | 0.479 |
|  | 1.16 | (0.55 – 1.71) | | 1.17 | (0.54 – 1.71) | | | 1.16 | | | (0.55 – 1.71) |  | | |
| Not reported | 1 | (0.1%) | | 0 | (-) | | | 1 | | | (0.1%) |  | | |
| HLA-A mismatches |  | |  |  | |  |  | |  | | | | 0.311 | |
| 0 | 187 | (15.6%) | | 65 | (12.8%) | | | 252 | | | (14.8%) |  | | |
| 1 | 707 | (59.1%) | | 312 | (61.3%) | | | 1019 | | | (59.8%) |  | | |
| 2 | 302 | (25.3%) | | 132 | (25.9%) | | | 434 | | | (25.5%) |  | | |
| HLA-B mismatches |  | |  |  | |  |  | |  | | | | 0.704 | |
| 0 | 55 | (4.6%) | | 22 | (4.3%) | | | 77 | | | (4.5%) |  | | |
| 1 | 571 | (47.7%) | | 233 | (45.8%) | | | 804 | | | (47.2%) |  | | |
| 2 | 570 | (47.7%) | | 254 | (49.9%) | | | 824 | | | (48.3%) |  | | |
| HLA-Cw mismatches |  |  | |  |  | | |  | | |  | 0.942 | | |
| 0 | 163 | (13.6%) | | 68 | (13.4%) | | | 231 | | | (13.6%) |  | | |
| 1 | 595 | (49.8%) | | 258 | (50.7%) | | | 853 | | | (50.0%) |  | | |
| 2 | 438 | (36.6%) | | 183 | (36.0%) | | | 621 | | | (36.4%) |  | | |
| HLA-DQ mismatches |  |  | |  |  | | |  | | |  | 0.248 | | |
| 0 | 408 | (34.1%) | | 185 | (36.4%) | | | 593 | | | (34.8%) |  | | |
| 1 | 705 | (59.0%) | | 299 | (58.7%) | | | 1004 | | | (58.9%) |  | | |
| 2 | 83 | (6.9%) | | 25 | (4.9%) | | | 108 | | | (6.3%) |  | | |
| HLA-DR mismatches |  |  | |  |  | | |  | | |  | 0.059 | | |
| 0 | 95 | (7.9%) | | 58 | (11.4%) | | | 153 | | | (9.0%) |  | | |
| 1 | 694 | (58.0%) | | 275 | (54.0%) | | | 969 | | | (56.8%) |  | | |
| 2 | 407 | (34.0%) | | 176 | (34.6%) | | | 583 | | | (34.2%) |  | | |
| HLA mismatch level | | | | | | | |  | | |  | 0.243 | | |
| Level 1 and 2 | 54 | (4.5%) | | 32 | (6.3%) | | | 86 | | | (5.0%) |  | | |
| Level 3 | 394 | (32.9%) | | 156 | (30.7%) | | | 550 | | | (32.3%) |  | | |
| Level 4 | 748 | (62.5%) | | 321 | (63.1%) | | | 1069 | | | (62.7%) |  | | |
| Percentages may not total 100% due to rounding. | | | | | | | | | | | | | | |

*Table S3 – Comparison of landmark and overall unadjusted 10-year pancreas graft survival estimates, by induction therapy*

| **Induction Therapy** | **10-year pancreas graft survival** | |
| --- | --- | --- |
|  | **1-month landmark**  **survival rate (95% CI)** | **Survival rate**  **(95% CI)** |
| Alemtuzumab | 79.7% (76.7 - 82.4%) | 74.6% (71.5 - 77.3%) |
| Basiliximab | 74.8% (70.0 - 79.0%) | 68.4% (63.6 - 72.6%) |
| **Log-rank p-value** | 0.050 | 0.012 |

*Table S4 – Sensitivity analysis comparing simple imputation and complete-case cohorts for 10-year pancreas graft survival.*

| **Factor** | **10-year pancreas graft failure** | | | | | | | |
| --- | --- | --- | --- | --- | --- | --- | --- | --- |
|  | **Simple imputation cohort**  **(n=1705)** | | | | **Complete-case cohort**  **(n=1575)** | | | |
|  | N | Observed  Events | HR (95% CI) | P*-*value | N | Observed Events | HR (95% CI) | P*-*value |
| Era of transplant |  |  |  | 0.038 |  |  |  | 0.022 |
| Apr 2007-Mar 2011 | 564 | 166 | Reference |  | 489 | 148 | Reference |  |
| Apr 2011-Mar 2015 | 612 | 143 | 0.81 (0.64 – 1.03) |  | 571 | 135 | 0.79 (0.61 – 1.01) |  |
| Apr 2015-Mar 2019 | 529 | 88 | 0.70 (0.52 – 0.93) |  | 515 | 85 | 0.67 (0.49 – 0.89) |  |
| Donor type |  |  |  | 0.36 |  |  |  | 0.46 |
| DBD | 1385 | 324 | Reference |  | 1268 | 298 | Reference |  |
| DCD | 320 | 73 | 1.14 (0.87 – 1.49) |  | 307 | 70 | 1.11 (0.84 – 1.46) |  |
| Donor age (years) | 1705 | 397 | 1.02 (1.01 – 1.03) | <0.0001 | 1575 | 368 | 1.02 (1.01 – 1.02) | 0.0002 |
| Donor CMV status |  |  |  | 0.03 |  |  |  | 0.07 |
| Negative | 984 | 214 | Reference |  | 904 | 200 | Reference |  |
| Positive | 721 | 183 | 1.25 (1.02 – 1.53) |  | 671 | 168 | 1.22 (0.99 – 1.51) |  |
| Recipient age (years) | 1705 | 397 | 0.97 (0.96 – 0.98) | <0.0001 | 1575 | 368 | 0.97 (0.96 – 0.98) | <0.0001 |
| Recipient blood group |  |  |  | 0.059 |  |  |  | 0.063 |
| O | 739 | 174 | Reference |  | 681 | 163 | Reference |  |
| A | 717 | 178 | 1.16 (0.94 – 1.43) |  | 664 | 164 | 1.13 (0.90 – 1.40) |  |
| B | 189 | 35 | 0.75 (0.52 – 1.08) |  | 172 | 32 | 0.72 (0.49 – 1.06) |  |
| AB | 60 | 10 | 0.70 (0.37 – 1.33) |  | 58 | 9 | 0.64 (0.32 – 1.25) |  |
| Sensitisation group |  |  |  | 0.22 |  |  |  | 0.19 |
| < 85% | 1625 | 382 | Reference |  | 1496 | 353 | Reference |  |
| ≥ 85% | 80 | 15 | 0.72 (0.42 – 1.22) |  | 79 | 15 | 0.70 (0.41 – 1.20) |  |
| Cold ischaemic time (hours) | 1705 | 397 | 1.05 (1.01 – 1.08) | 0.015 | 1575 | 368 | 1.04 (1.01 – 1.08) | 0.025 |
| HLA-A mismatches |  |  |  | 0.65 |  |  |  | 0.98 |
| 0 | 252 | 51 | Reference |  | 235 | 51 | Reference |  |
| 1 | 1019 | 235 | 1.11 (0.81 – 1.51) |  | 938 | 217 | 1.03 (0.75 – 1.40) |  |
| 2 | 434 | 111 | 1.18 (0.84 – 1.65) |  | 402 | 100 | 1.04 (0.73 – 1.47) |  |
| HLA-B mismatches |  |  |  | 0.008 |  |  |  | 0.012 |
| 0 | 77 | 17 | Reference |  | 73 | 15 | Reference |  |
| 1 | 804 | 204 | 1.25 (0.74 – 2.11) |  | 746 | 190 | 1.38 (0.79 – 2.40) |  |
| 2 | 824 | 176 | 0.89 (0.52 – 1.53) |  | 756 | 163 | 0.99 (0.56 – 1.76) |  |
| HLA-Cw mismatches |  |  |  | 0.14 |  |  |  | 0.11 |
| 0 | 231 | 47 | Reference |  | 212 | 43 | Reference |  |
| 1 | 853 | 197 | 1.22 (0.88 – 1.71) |  | 790 | 181 | 1.20 (0.85 – 1.71) |  |
| 2 | 621 | 153 | 1.42 (0.99 – 2.03) |  | 573 | 144 | 1.45 (1.00 – 2.11) |  |
| HLA-DQ mismatches |  |  |  | 0.033 |  |  |  | 0.045 |
| 0 | 593 | 163 | Reference |  | 553 | 153 | Reference |  |
| 1 | 1004 | 205 | 0.75 (0.59 – 0.94) |  | 924 | 189 | 0.74 (0.59 – 0.94) |  |
| 2 | 108 | 29 | 0.94 (0.60 – 1.47) |  | 98 | 26 | 0.90 (0.56 – 1.44) |  |
| HLA-DR mismatches |  |  |  | 0.52 |  |  |  | 0.79 |
| 0 | 153 | 41 | Reference |  | 143 | 40 | Reference |  |
| 1 | 969 | 227 | 1.10 (0.76 – 1.59) |  | 897 | 207 | 1.03 (0.71 – 1.5) |  |
| 2 | 583 | 129 | 0.97 (0.64 – 1.46) |  | 535 | 121 | 0.94 (0.62 – 1.43) |  |
| Induction agent |  |  |  | 0.91 |  |  |  | 0.76 |
| Alemtuzumab | 1196 | 256 | Reference |  | 1098 | 239 | Reference |  |
| Basiliximab | 509 | 141 | 0.98 (0.66 – 1.45) |  | 477 | 129 | 0.94 (0.62 – 1.42) |  |

Table S5 – Interaction term, HLA-DQ mismatch × induction therapy, for 10-year pancreas graft survival*

| **Interaction: HLA-DQ mismatch × induction therapy** | **Hazard Ratio**  **(95% CI)** | **p-value** |
| --- | --- | --- |
| DQ mismatch 1 vs 0 with Alemtuzumab | 0.73 (0.56 – 0.96) | 0.71 |
| DQ mismatch 2 vs 0 with Alemtuzumab | 0.84 (0.50 – 1.43) |  |
| DQ mismatch 1 vs 0 with Basiliximab | 0.77 (0.54 – 1.11) |  |
| DQ mismatch 2 vs 0 with Basiliximab | 1.21 (0.58 – 2.55) |  |

**Adjusted for transplant era, donor type, donor age, donor CMV, recipient age, recipient blood group, sensitisation group, cold ischaemic time, mismatches at HLA-A, -B, -Cw, -DQ, -DR and induction therapy.*
